# Pilonidal sinus of breast: A case report with literature review

**DOI:** 10.1016/j.ijscr.2019.11.057

**Published:** 2019-12-09

**Authors:** Abdulwahid M. Salih, Fahmi H. Kakamad, Zuhair D. Hammood, Hiwa O. Baba, Imad J. Habibullah, Rawezh Q. Salih, Shvan H. Mohammed

**Affiliations:** a Faculty of Medical Sciences, School of Medicine, Department Surgery, University of Sulaimani, Sulaimani, Kurdistan, Iraq; b Faculty of Medical Sciences, School of Medicine, Department Cardiothoracic and Vascular Surgery, University of Sulaimani, Sulaimani, Kurdistan, Iraq; c Kscien Organization, Hamdi Str., Azadi Mall, Sulaimani, Kurdistan, Iraq; d Sulaimani Teaching Hospital, Sulaimani, Kurdistan, Iraq; e Shar Medical Center, Laboratory Department, Ibrahim Pasha Street, Sulaimani, Kurdistan, Iraq; f Chara Laboratory, Shahedan Street, Kalar, Kurdistan, Iraq

**Keywords:** Breast, Mammary, Pilonidal, Sinus, Abscess, Case report

## Abstract

**Introduction:**

Breast pilonidal sinus is an extremely rare variant of the condition, the report aims to present a case of pilonidal sinus occurring in the breast.

**Case presentation:**

A 35-year-old female presented with a left breast discharging lesion for a 5-month duration. There was a local redness with multiple discharging sinuses on her left breast at 3–5 o’clock, 3–4 cm away from the nipple. The discharge was serosanguinous in appearance. The lesion was excised totally with primary closure under general anesthesia. Histopathological examination was consistent with pilonidal sinus.

**Discussion:**

For atypical pilonidal sinus disease, surgery is the modality of choice but regarding sacrococcygeal pilonidal sinus, non-operative treatment begins to grow. The cardinal risk factors for recurrence and complications are male gender, family history, tobacco smoking, obesity, size of sinus, poor personal hygiene and surgical methods.

**Conclusion:**

Breast pilonidal sinus is an extremely rare variant of the condition. It should be suspected on clinical examination. Surgical excision is the definitive treatment strategy.

## Highlights

• Sacrococcygeal area is the commonest site of PNS.
• However, it could be found in other regions like hand, intermammary, suprapubic, umbilicus, nose etc.
• Breast pilonidal sinus is an extremely rare variant of PNS.
• The aim of this report is to present a case of pilonidal sinus occurring in breast.

## 1 Introduction

One of the common medical situations that accounts for almost 15% of suppurative anal conditions is pilonidal Sinus (PNS). It is an inflammatory condition resulting from skin penetration by a hair. The tract of the sinus is lined by granulation tissue which terminates in a pus-filled chamber [1,2]. The typical age range of affected individuals is 10–40 years [3]. Sacrococcygeal area is the commonest site of PNS. however, it could be found in other regions like the hands, intermammary, suprapubic, umbilicus, nose, interdigital web, groin, face, neck, prepuce, penis, postauricular, preauricular, submental, clitoris, scalp, endoanal, and axilla [[4], [5], [6], [7], [8], [9], [10]]. It may occur as an opened and discharging tract or as a manifestation of repetitive pus collection that presented like an inflammatory pattern: redness, local pain, warmth and tenderness [11,12].

Breast PNS (bPNS) is an extremely rare variant of the condition with only three reported cases in the literature [2,12,13]. The paper aims to report a rare case of bPNS in line with SCARE guidelines with a brief review of the condition [14].

### 1.1 Patient information

A 35-year-old housewife presented with a discharging lesion on her left breast for 5 months, not responding to medical treatment with a history of remission and relapse. She was nonsmoker, multiparous. Past-history was unremarkable.

### 1.2 Clinical findings

**Fig. 1:**
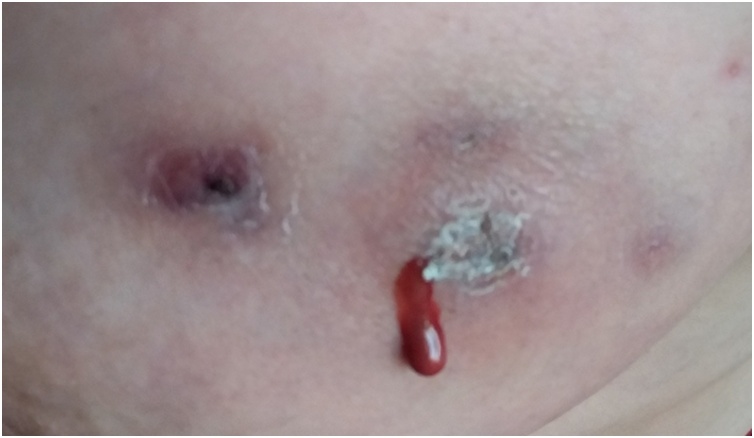
Multiple discharging sinuses on her left breast at 3–5 o’clock.

There was a local redness with multiple discharging sinuses on her left breast at 3–5 o’clock, 3–4 cm away from the nipple (Fig. 1). The discharge was mucoid in appearance.

### 1.3 Therapeutic intervention

**Fig. 2:**
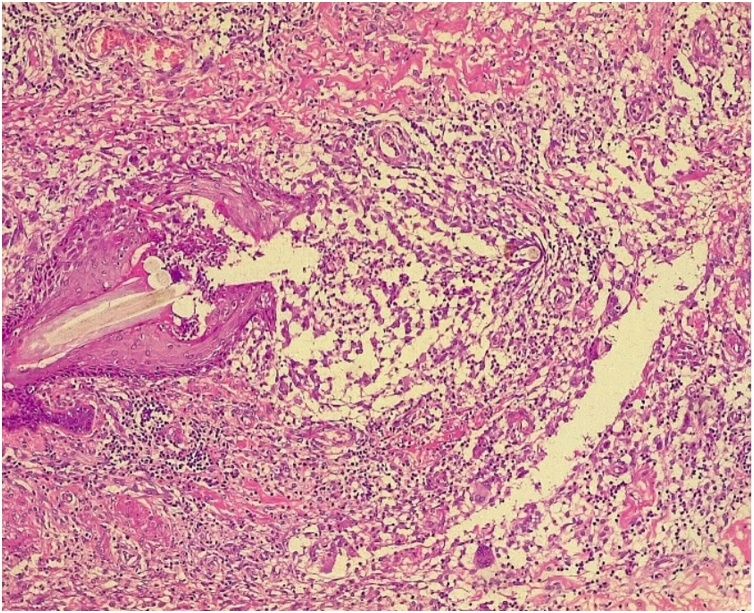
Hair shaft surrounded by granulation tissue.

The lesion was excised totally with primary closure under general anesthesia. Histopathological examination showed invaginated epidermis forming a tract extending from the subcutaneous tissue to the skin. There was a hair shaft surrounded by granulation, consistent with a pilonidal sinus (Fig. 2).

### 1.4 Follow-up and outcomes

Postoperatively she was followed up for six months, the wound healed completely without complications and there was no evidence for recurrence.

## 2 Discussion

Before puberty and after the age of forty, PNS is uncommon. The condition demonstrates male dominance with a ratio of 3:1 [15].

Young age, deep navel and cleft, hairiness, male sex, long term sitting and poor personal hygiene are among the risk factors [3]. In the management of sPNS, a variety of surgical and noninvasive techniques have been investigated. Drainage and simple incision, lying open, marsupialization, primary closure and excision, or rhomboid excision and Limberg flap were included in operative management while non-operative methods consist of different wound enhancing solutions and sclerosing agents that injected into the tract [16,17]. The ideal management should be complication-free, effective and safe, also it should minimize patient’s discomfort, recurrence rate, complications, hospital stay and long work absence. Surgical methods put the patient at risk of discomfort and long duration loss of workdays, so they do not meet the ideal treatment strategies. For atypical PNS, surgery is the modality of choice but regarding sPNS non-operative treatment begins to grow [3,17]. Recurrence and complications are not uncommon. According to the studies, cardinal risk factors for recurrence and complications are male gender, family history, tobacco, obesity, size of sinus, poor personal hygiene and surgical methods.

bPNS is a very rare variant of PNS [2,12,13]. Keighley et al. received a 52-year-old lady presenting with periareolar pain, and nipple inversion. Clinical assessment and ultrasound examination revealed periareolar abscess resistant to medication and frequent aspiration. They performed surgical excision. Histopathological examination confirmed the diagnosis of bPNS [2].

Lahiri and associates presented a case of bPNS occurred in a 28-year-old female. She presented with a 4-week history of left breast collection, the condition was provisionally diagnosed as an abscess complicating periductal mastitis, surgical excision was done and microscopic examination of the specimen revealed features consistent with bPNS [12].

Huges et al. reported their experience with a 38-year-old male patient who presented with a right breast mass for a 20-year duration with intermittent discharge from the lesion. After a thorough clinical examination and ultrasound study, they excised the lesion, histopathological examination demonstrated peri-areolar PNS disease [13].

In composite, bPNS is an extremely rare variant of the condition. It should be suspected on clinical examination. Surgical excision is the definitive treatment strategy.

## Sources of funding

No source to be stated.

## Ethical approval

Approval has been taken from Kscien centre.

## Consent

Consent has been taken from the patient and the family of the patient.

## Author’s contribution

Abdulwahid M. Salih: Surgeon performed the operation and follow up.

Shvan H. Mohammed, Fahmi H. Kakamad, and Rawezh Q. Salih: Writing the manuscript and follow up.

Zuhair D. Hammood, Hiwa O. Baba, and Imad J. Habibullah: literature review, final approval of the manuscript.

## Registration of research studies

Not applicable.

## Guarantor

Fahmi Hussein kakamad.

## Provenance and peer review

Not commissioned, externally peer-reviewed.

## Declaration of Competing Interest

There is no conflict to be declared.
